# Olive seed protein bodies store degrading enzymes involved in mobilization of oil bodies

**DOI:** 10.1093/jxb/ert355

**Published:** 2013-10-29

**Authors:** Agnieszka Zienkiewicz, Krzysztof Zienkiewicz, Juan David Rejón, Juan de Dios Alché, Antonio Jesús Castro, María Isabel Rodríguez-García

**Affiliations:** ^1^Department of Biochemistry, Cell and Molecular Biology of Plants, Estación Experimental del Zaidín, CSIC, 18008 Granada, Spain; ^2^Chair of Plant Physiology and Biotechnology, Nicolaus Copernicus University, 87 - 100 Toruń, Poland; ^3^Department of Cell Biology, Nicolaus Copernicus University, 87 - 100 Toruń, Poland

**Keywords:** Lipase, lipoxygenase, oil bodies, olive, phospholipase A, protein bodies.

## Abstract

The major seed storage reserves in oilseeds are accumulated in protein bodies and oil bodies, and serve as an energy, carbon, and nitrogen source during germination. Here, the spatio-temporal relationships between protein bodies and several key enzymes (phospholipase A, lipase, and lipoxygenase) involved in storage lipid mobilization in cotyledon cells was analysed during *in vitro* seed germination. Enzyme activities were assayed in-gel and their cellular localization were determined using microscopy techniques. At seed maturity, phospholipase A and triacylglycerol lipase activities were found exclusively in protein bodies. However, after seed imbibition, these activities were shifted to the cytoplasm and the surface of the oil bodies. The activity of neutral lipases was detected by using α-naphthyl palmitate and it was associated mainly with protein bodies during the whole course of germination. This pattern of distribution was highly similar to the localization of neutral lipids, which progressively appeared in protein bodies. Lipoxygenase activity was found in both the protein bodies and on the surface of the oil bodies during the initial phase of seed germination. The association of lipoxygenase with oil bodies was temporally correlated with the appearance of phospholipase A and lipase activities on the surface of oil bodies. It is concluded that protein bodies not only serve as simple storage structures, but are also dynamic and multifunctional organelles directly involved in storage lipid mobilization during olive seed germination.

## Abstract (precis)

New evidence on a spatio-functional relationship between protein bodies (PBs) and oil bodies (OBs) during olive seed germination. PBs do not only store the key enzymes required for lipid breakdown but are also directly involved in this process

## Introduction

Seed germination is accompanied by intense metabolic activity of cells, including respiration and protein synthesis, as well as multiple cell divisions, cell elongation, and complex structural changes at the subcellular level. Establishment of the seedling is the result of proper seed germination, and seedling growth is initially supported by metabolites produced by the hydrolysis and conversion of the major stored seed reserves, such as proteins and lipids (Bewley and Black, 1994; Zienkiewicz *et al.*, 2011).

Seed storage proteins (SSPs) are degraded during germination and used for seedling growth. These proteins provide carbon, nitrogen, and sulphur resources for subsequent seedling development (Müntz, 1996; Herman and Larkins, 1999). SSPs are accumulated during seed maturation in the protein storage vacuoles (PSVs) of the embryo and endosperm cells. At the late stage of seed maturation, the PSVs are transformed into protein bodies (PBs), which are surrounded by a membrane derived from the vacuolar membrane (Hara-Nishimura *et al.*, 1987; Strzałka *et al.*, 1995). Seed storage triacylglycerols (TAGs) are accumulated in the embryo and endosperm during seed development and also provide the sources of carbon and energy for germination and post-germinative growth of seedlings (Napier *et al.*, 1996). The storage lipids are assembled in discrete spherical organelles of 0.5–2.5 μm called oil bodies (OBs) or lipid bodies (Murphy, 2012). The OB consists of a core of TAGs covered by a single layer of phospholipids with proteins embedded. These proteins (oleosin, caleosin, and steroleosin) are responsible for the stability of OBs and prevent the degradation of storage lipids until the seed germinates (Tai *et al.*, 2002; Lin and Tzen, 2004; Poxleitner *et al.*, 2006). Storage lipid mobilization is initiated by the activity of endogenous lipases, which hydrolyse storage TAGs to glycerol and free fatty acids (Quettier and Eastmond, 2009). *SUGAR-DEPENDENT* (*SDP1*) and *SDP1-LIKE* (*SDP1L*) encode a TAG lipase with a patatin-like acyl-hydrolase domain that can associate with the OB surface and is capable of hydrolysing TAGs in preference to diacylglycerol or monoacylglycerol (Eastmond, 2006; Kelly *et al.*, 2011). In a alternative pathway for TAG mobilization, a specific lipoxygenase (LOX) enzyme is involved. Matsui *et al.* (1999) found that trypsin *in vitro* digestion of OB-associated proteins led to the oxygenation of TAGs by the action of LOX in cucumber cotyledons. This lipoxygenase is capable of catalysing stereospecific oxygenation of the linoleate moieties of TAGs to (9*Z*,11*E* 1,2*Z*)-13-hyroperoxy octadeca-9,11-dienoic acid (13-HPOD) (Feussner and Wasternack, 1998; Gerhardt *et al.*, 2005). More recently, it was reported that a patatin-like phospholipase (PLA) promotes the LOX-dependent oxygenation of OB phospholipids in cucumber cotyledons (Rudolph *et al.*, 2011).

Seed OBs have often been shown to associate with other organelles such as glyoxysomes, PSVs, or PBs (Fernandez and Staehelin, 1987; Hayashi *et al.*, 2001; Poxleitner *et al.*, 2006). There is agreement that this interaction could help breach the OB membrane in order to trigger the process of TAG degradation (Hayashi *et al.*, 2001; Poxleitner *et al.*, 2006). A direct relationship between OBs and glyoxysomes is thought to facilitate fatty acid release and their transfer between the two organelles. Moreover, this process requires the participation of a lipase active at neutral pH (Hayashi *et al.*, 2001). Other data suggested that PBs store lipases in their membranes; thus, one of the possible functions of the OB–PB association might be to join the enzyme and its substrate (Fernandez and Staehelin, 1987). Previous results showed that in mature olive seed the cotyledon cells contain large PBs surrounded by numerous OBs (Alché *et al.*, 2006; Zienkiewicz *et al.*, 2011). During seed germination, a progressive decrease of OB and PB number was observed, which is accompanied by a close spatial relationship between these two organelles (Zienkiewicz *et al.*, 2011).

To date, despite extensive studies of TAG mobilization during seed germination, little is known about the nature of PB–OB interaction. Here, new evidence is reported on the direct involvement of PBs in breakdown of OBs during seed germination. By analysing the spatio-temporal behaviour of lipase, LOX, and PLA, it was demonstrated that PBs are spatially and functionally connected to storage lipid mobilization.

## Materials and methods

### Plant material

Mature seeds of *Olea europaea* L. were obtained from olive trees (cv. ‘Picual’) grown in the Estación Experimental del Zaidín (Granada, Spain).

### *In vitro* germination of olive embryos

*In vitro* germination of olive embryos was carried out as described by Cañas and Benbadis (1988). Cotyledons were collected from mature and imbibed (24h) seeds and at different times of *in vitro* germination (6h and 3 d) and seedling growth (4, 8, 15, and 26 d).

### RNA isolation and cDNA synthesis

Frozen samples were ground in liquid nitrogen using a mortar and pestle. Total RNA was extracted using an RNeasy Plant Total RNA kit (Quiagen, Germany). First-strand cDNA was synthesized with 0.5 μg total of RNA, oligo(dT)_19_ primer (0.5 μg), and reverse transcriptase (Fermentas, Germany) according to the manufacturer’s instructions.

### Quantitative real-time PCR (qRT-PCR)

Gene expression analysis was performed by qRT-PCR using an iCycler (Bio-Rad, USA). Primers for gene-specific amplification (Supplementary Table S1 available at *JXB* online) were designed using the Primer3 program (http://frodo.wi.mit.edu/cgi-bin/primer3/primer3_www.cgi). Target genes (and the olive ubiquitin2 gene as a housekeeping marker; Padilla *et al.*, 2012) were subjected to qRT-PCR in 96-well optical reaction plates in 20 μl mixtures per well, using iQ™ SYBR^®^ Green Supermix (Bio-Rad) and following the manufacturer’s instructions. Cycle threshold (Ct) values were obtained with the iQ™ software (Bio-Rad) and data were analysed with the 2^ΔΔCT^ method (Livak and Schmittgen, 2001). The data are presented as means ±standard deviation (SD) of three biological repeats, obtained from three independent experiments.

### Isolation of protein and oil bodies

PBs and OBs were isolated from cotyledons at different times of *in vitro* germination according to Torrent *et al.* (1989) and Zienkiewicz *et al.* (2010), respectively.

### Protein extraction

Material was powdered in liquid nitrogen and suspended in 1.5ml of extraction buffer (0.05M phosphate buffer, pH 7.0). Total proteins and proteins from the PB fraction were eluted under continuous and vigorous stirring at 4 °C for 2h. The samples were then centrifuged at 13 500 *g* for 30min at 4 °C and the resulting supernatants were used for activity assays and Western blot analysis. OB-associated proteins were extracted as described by Zienkiewicz *et al.* (2010). Protein content in each extract was measured by using a commercial Bradford procedure (Bio-Rad).

### SDS–PAGE and immunoblotting

SDS–PAGE was performed according to Laemmli (1970) on 12% (w/v) acrylamide gels with 4.5% stacking gels. Total proteins (50 μg per sample) were mixed with an equal volume of 2× SDS sample buffer (Laemmli, 1970) and boiled for 3min prior to gel loading. After electrophoresis, the resulting gels were stained by Coomassie Brilliant blue (CBB), or were transferred onto PVDF membranes in a Semi-dry Transfer Cell (Bio-Rad). The membranes were blocked in Tris-buffered saline (TBS) buffer containing 0.5% (w/v) non-fat dry milk for 1h. Immunodetection of LOX was carried out by incubation with a polyclonal anti-*Glycine max* (soybean) LOX antibody (Ab) (Agrisera, Sweden) diluted 1:1000 in TBS buffer for 12h at 4 °C. A DyLight 488 conjugated anti-rabbit IgG (Agrisera), diluted 1:2000 in TBS buffer for 2h, served as the secondary Ab. The signal was detected in a Pharos FX molecular imager (Bio-Rad). Densitometric measurements were carried out from images of membranes using Quantity One 4.6.2 software (Bio-Rad).

### In-gel lipase and LOX activity

SDS–PAGE was performed as above, but the sample boiling step was omitted. After electrophoresis, SDS was removed from polyacrylamide gels by washing them three times for 30min each in a solution containing 0.05M phosphate buffer (pH 7.0) and 2.5% (v/v) Triton X-100. Lipase activity was measured as previously described (Rejón *et al.*, 2012). LOX activity assays were prepared according to Heinish *et al.* (1996). The gel was incubated for 30min in 50ml of solution containing 0.2M borate buffer (pH 9.0) and 50 μl of α-linolenic acid (dissolved in 50 μl of ethanol). Subsequently, the gel was rinsed briefly in distilled water and incubated with 100ml of a solution containing 0.5g of *N*,*N*-dimethyl-*p*-phenylenediamine, 4.5ml of methanol, and 0.5ml of acetic acid.

### Histochemical studies

Material was processed for light microscopy as previously described (Zienkiewicz *et al.*, 2011). Protein material and neutral lipids were stained according to Serrano *et al.* (2008). The samples were observed in a LM Zeiss Axioplan microscope (Carl Zeiss, Germany) and images were obtained with a ProGres C3 digital camera using the ProGres CapturePro 2.6 software (Jenoptik AG, Germany).

### Microscopic immunolocalization of LOX

Semi-thin sections were incubated overnight at 4 °C with an anti-LOX Ab [diluted 1:50 in 1% bovine serum albumin (BSA) in phosphate-buffered saline (PBS) pH 7.2], following by an anti-rabbit IgG DyLight 488-conjugated secondary Ab (diluted 1:200 in 1% BSA in PBS pH 7.2) for 1h at 37 °C. Samples were observed in a Zeiss Axioplan microscope using the filter combination: BP365, FT395, and LP397. For immunogold experiments, ultra-thin sections were incubated with an anti-LOX Ab (diluted 1:50 in 1% BSA in PBS pH 7.2), overnight at 4 °C, followed by an anti-rabbit IgG 15nm gold-conjugated secondary Ab (British Biocell International, UK) diluted 1:100 in 1% BSA in PBS pH 7.2 for 1h. After washing, sections were stained with 5% (w/v) uranyl acetate for 30min. Samples were observed in a JEM-1011 transmission electron microscope (JEOL, Japan) operating at 80kV. Negative controls were prepared by omitting the incubation with the primary Ab or by incubation with a pre-immune rabbit serum.

### Light microscopic localization of lipase and LOX activity

Lipase activity was revealed by incubating the semi-thin sections for 30min at 37 ºC in a developing solution containing 40mg of α-naphthyl palmitate prepared in 16ml of *N*,*N*-dimethylformamide and 80mg of Fast blue BB salt in 144ml of 0.1M phosphate buffer, pH 7.0. LOX activity was detected by incubating the semi-thin sections for 30min at room temperature in a solution containing α-linolenic acid. Then, the sections were stained with a solution containing 0.5g of *N*,*N*-dimethy-*p*-phenylenediamine, 4.5ml of methanol, and 0.5ml of acetic acid. Observations were carried out with an Axioplan microscope.

### *In situ* localization of lipase and PLA activity

For whole-mount localization, olive cotyledons were cut into small pieces using a scalpel. Detection of neutral lipids was carried out by using Nile Red. Samples were incubated for 10min in a solution containing 0.05mg ml^–1^ Nile Red (Sigma-Aldrich) dissolved in acetone. Samples were observed with a Nikon C1 confocal laser scanning microscope (CLSM) (Nikon, Japan) using an argon (488) laser. Z-series images were collected and processed with the software EZ-C1 Gold version 2.10 build 240 (Nikon). Detection of cellular membranes was performed by using FM4-64 dye according to the manufacturer’s instructions (Molecular Probes, USA). SYTOX green nucleic acid stain was used for determination of cotyledon cell viability and was performed according to the manufacturer’s instructions (Molecular Probes). Heat treatment (95 °C for 10min) was used as a positive control of cells with permeabilized membranes. In order to detect lipase activity, samples were incubated for 30min in an aqueous solution of 25 μg ml^–1^ resorufin ester (lipase substrate, Sigma-Aldrich). Samples were observed with a Nikon C1 CLSM using a He–Ne (549nm) laser. Phospholipase A_1_ and A_2_ activities were detected using BODIPY^®^ FL C_11_-PC (Molecular Probes). Samples were incubated for 10min in 1mM BODIPY^®^ FL C_11_-PC ethanolic solution and observed with a Nikon C1 CLSM using an argon (488nm) laser. *In situ* localization of lipase and PLA activity was also performed in isolated OBs as described previously by Zienkiewicz *et al.* (2013)

### Immunolocalization experiments in isolated OBs

Purified OBs were incubated in a 1.5ml tube with the anti-LOX Ab, rabbit anti-ubiquitin (Alché *et al.*, 2000), or the mixture of anti-LOX and chicken anti-Clo (Davids Biotechnologie, Germany) Abs (diluted 1:30 in PBS buffer, pH 7.2, containing 1% BSA), for 2h at room temperature, followed by incubation with an anti-rabbit IgG DyLight 550-conjugated secondary Ab (Agrisera), and/or an anti-chicken IgY DyLight 448-conjugated secondary Ab (Agrisera) diluted 1:200 in PBS buffer, pH 7.2 for 1h at 37 ºC under gentle agitation. Samples were observed with a Zeiss Axioplan epifluorescence microscope under blue and/or green light irradiation. Negative controls were treated as above, but the primary Ab was omitted or pre-immune serum was used instead of the primary Ab.

## Results

### In-gel detection of lipase activity

**Fig. 1.**
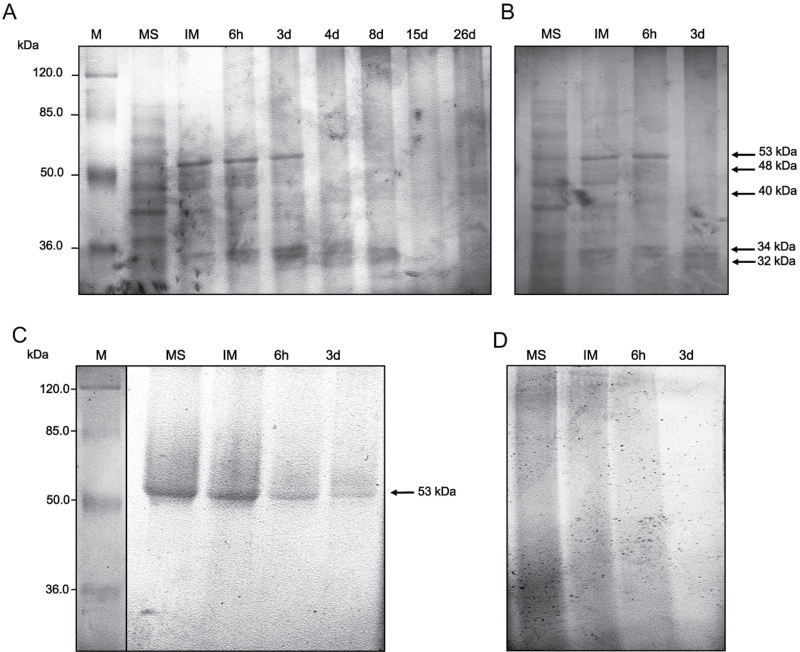
In-gel assay of lipase activity in the total protein extract (A) and in proteins associated with the cytoplasmic (B), PB (C), and OB (D) fractions. Major activity bands are indicated with arrows. M, protein markers; MS, mature dry seed; IM, seed after imbibition (24h); 6h, and 3 d, time of *in vitro* germination; 4 d, 8 d, 15 d, and 26 d, days of seedling growth.

Lipase activity was detected after protein separation on an SDS–polyacrylamide gel and after incubation with α-naphthyl palmitate as the substrate (Fig. 1A–D). At seed maturity, up to seven different lipolytic bands were observed in the total (Fig. 1A) and cytoplasmic fraction (Fig. 1B), with molecular weights ranging from 90kDa to 30kDa. After seed imbibition, only the lipolytic bands of 53, 48, 40, 34, and 32kDa were visible in both fractions (Fig. 1A, B, arrows). The band of 53kDa showed a higher intensity compared with the other bands and was detectable until the third day of *in vitro* germination, whereas the band of 40kDa was visible during the whole course of *in vitro* germination and seedling growth. In PB extracts, only one lipolytic band of 53kDa was detected during the course of *in vitro* germination (Fig. 1C). No lipolytic bands were detected in the OB fraction (Fig. 1D).

### Expression and activity of LOXs

**Fig. 2.**
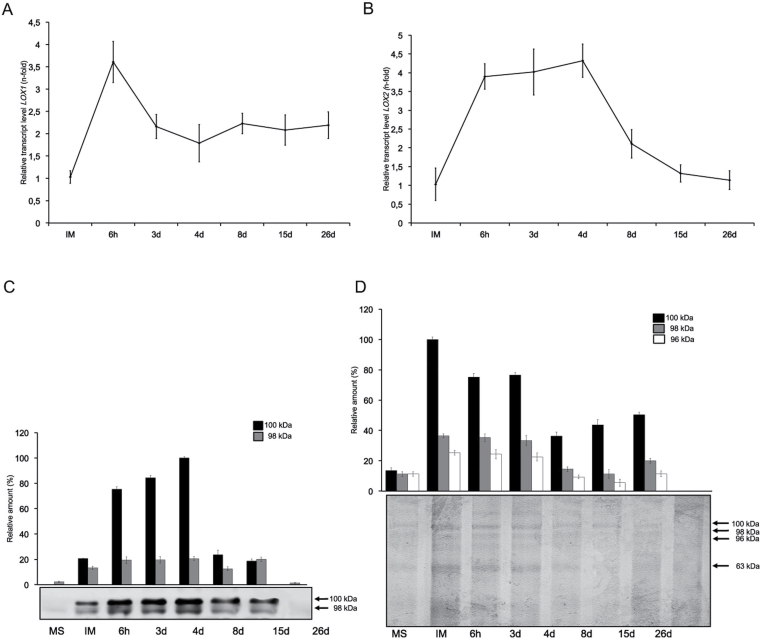
Relative expression levels of *OeLOX1* (A) and *OeLOX2* (B) determined by qRT-PCR using the expression level of the genes in mature seed cotyledons as the calibrator. (C) Immunodetection of lipoxygenase (arrows). (D) In-gel assay of lipoxygenase activity (arrows). MS, mature dry seed; IM, seed after imbibition (24h); 6h and 3d, time of *in vitro* germination; 4 d, 8 d, 15 d, and 26 d, days of seedling growth.

*OeLOX1* and *OeLOX2* mRNA levels were found to increase significantly after seed imbibition. The highest level of *OeLOX1* was observed at 6h of *in vitro* germination and was followed by a decrease after 3 d of *in vitro* germination (Fig. 2A). During the next stages of seedling growth, *OeLOX1* mRNA levels remained unchanged. High levels of *OeLOX2* transcripts were observed until the fourth day of seedling growth (Fig. 2B). A significant decrease in *OeLOX2* mRNA levels occurred at the final analysed steps of seedling growth. Western blot experiments showed the presence of two bands of ~100kDa and 98kDa, which were cross-recognized by the anti-LOX Ab (Fig. 2C). The specificity of this Ab was demonstrated previously (Zienkiewicz *et al.*, 2013). The LOX levels significantly increased after seed imbibition and during the subsequent steps of *in vitro* germination (Fig. 2C). No LOX proteins could be detected at the last analysed stage of seedling growth. In-gel assays of LOX activity indicated the presence of three LOX isozymes of ~100, 98, and 96kDa (Fig. 2D). The highest LOX activities were observed just after seed imbibition and during the first days of *in vitro* germination. Later steps of seedling growth were accompanied by a gradual decrease in all LOX activities (Fig. 2D).

### Distribution of total proteins, neutral lipids, and lipase activity in olive cotyledons

**Fig. 3.**
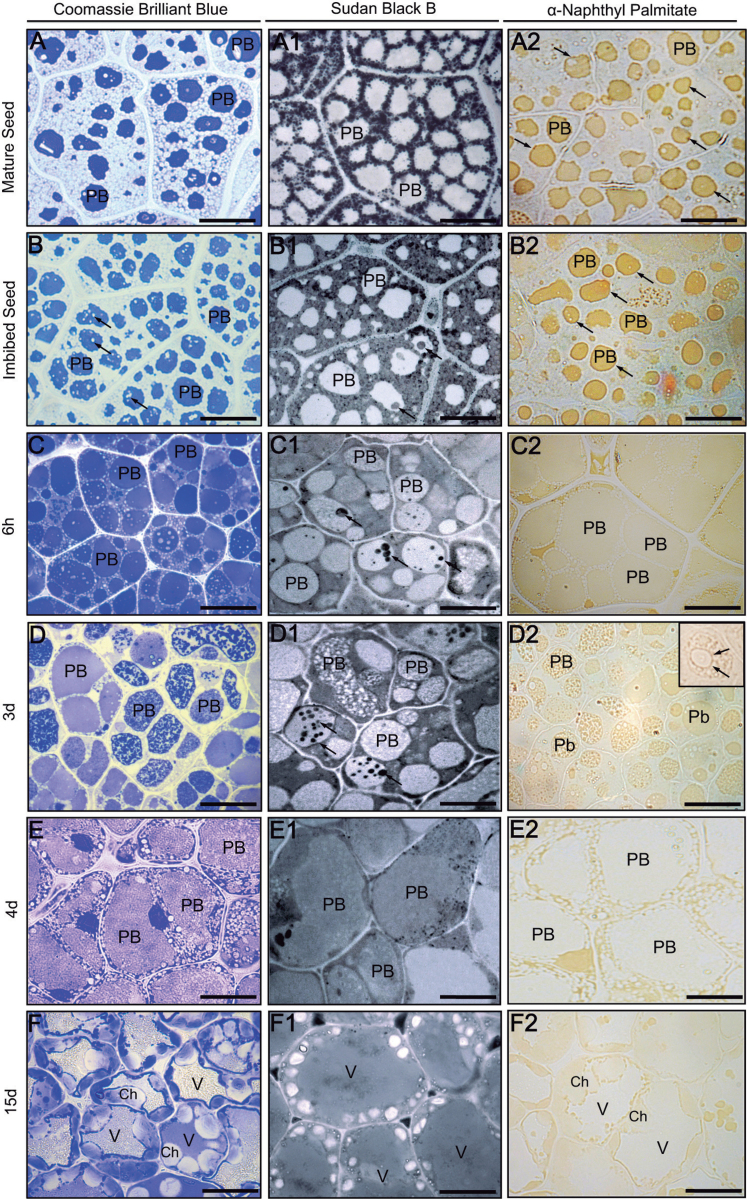
(A–F) Coomassie Brilliant blue (CBB) staining of total proteins in sections from olive cotyledons. (A1–F1) Sudan black B (SBB) staining of neutral lipids in sections from olive cotyledons. The arrows denote lipidic masses localized in the protein bodies. (A2–F2) Cellular localization of lipase activity in sections from olive cotyledons. Ch, chloroplast; PB, protein body; V, vacuole. Bars=25 μm.

CBB and Sudan Black B (SBB) stains were used to study the distribution of proteins and storage lipids in olive cotyledons, respectively. To analyse the potential correlation in the distribution of proteins, lipids, and lipase activity, α-naphthyl palmitate was used as a substrate. In mature seeds, proteins were detected in the area of PBs (Fig. 3A), while storage lipids were accumulated in the form of numerous OBs located around PBs (Fig. 3A1). At this stage, lipase activity was detected mainly in PBs, with a high concentration on their boundaries (Fig. 3A2, arrows). After seed imbibition, protein-free areas appeared in the PB matrix (Fig. 3B, arrows). In some PBs, rounded areas stained by SBB were also found (Fig. 3B1, arrows). Lipase activity was detected mainly in the area of PBs (Fig. 3B2, arrows). After 6h of *in vitro* germination, a rich pool of proteins was observed in the area of PBs and in the cytoplasm (Fig. 3C). SSB-stained areas in the matrix of the PBs were more numerous and more intensively stained when compared with imbibed seed (Fig. 3C1, arrows). Lipase activity was detected in the area of PBs as well as in the cytoplasm surrounding the OBs (Fig. 3C2). After 3 d of *in vitro* germination, proteins were uniformly distributed in some PBs, whereas in others they were visible as irregular clusters (Fig. 3D). Lipids were observed in the area of PBs in the form of irregular clusters and also as rounded areas after SSB staining (Fig. 3D1, arrows). The lipase activity was found in the matrix of the PBs, especially concentrated around lipid-containing PB areas (Fig. 3D2, insert). On the fourth day of seedling growth, when in most of the cotyledon cells only one, large PB was present, proteins and lipids were found in both the cytoplasm and PB matrix (Fig. 3E, E1). Lipase activity was detected mainly in the cytoplasm surrounding the central PB (Fig. 3E1). After 15 d of seedling growth, cells displayed differentiating chloroplasts, and proteins were detected in the chloroplast, cytoplasm, and the lumen of the central vacuole (Fig. 3F). Lipids were found homogenously distributed in the lumen of the central vacuole and in the cytoplasm (Fig. 3F1). Lipase activity was located mainly in the cytoplasm surrounding the central vacuole. Less intense staining was also observed in the chloroplasts (Fig. 3F2). Control reactions were performed by omitting lipase substrate and did not show any labelling (Supplementary Fig. S1A at *JXB* online).

### Localization of LOX proteins and activity in olive cotyledons

**Fig. 4.**
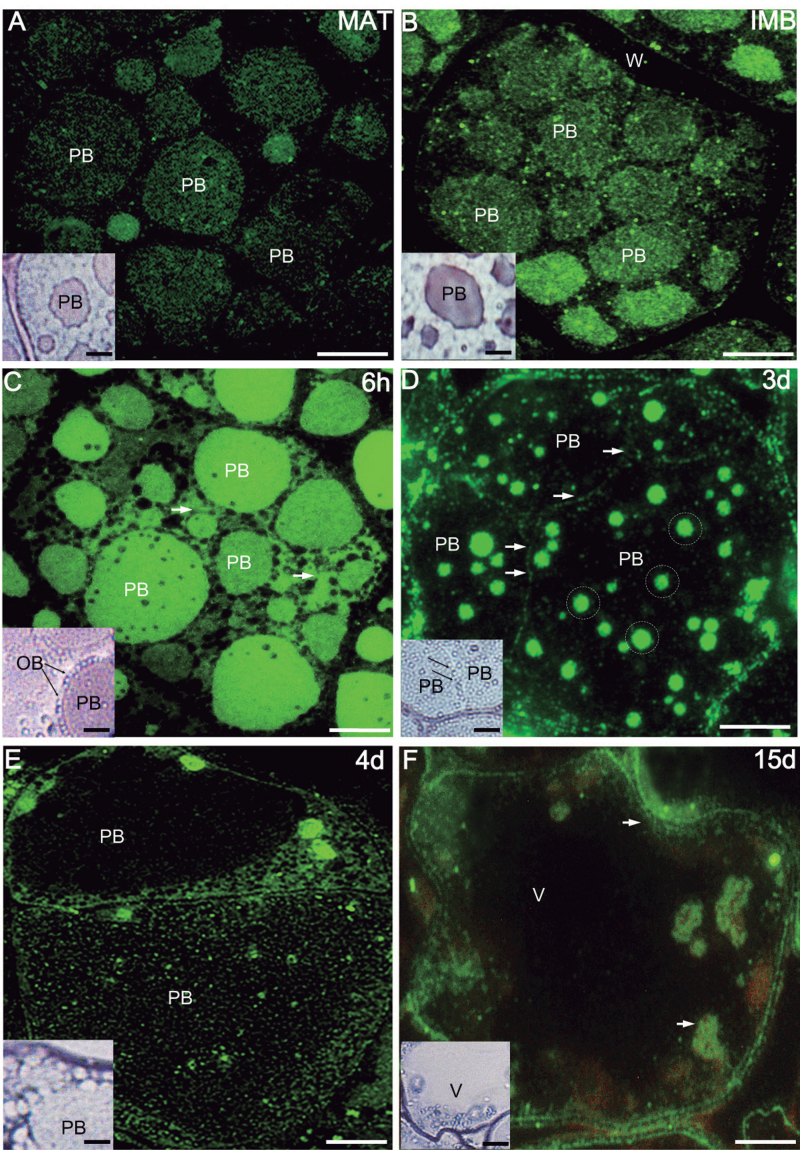
Localization of lipoxygenases (anti-LOX Ab) (A–F) with their activity (A–F, insets) in sections from olive cotyledons. Fluorescent labelling of LOX is located mainly in the area of PBs. After 6h of *in vitro* germination, the fluorescence appears in the OB boundary (arrows) and in the cytoplasm. An intense LOX activity is found at the boundaries of PBs connected with OBs. Significant LOX activity is observed on the surface of the OBs (insets, arrows). Bars=10 μm.

The cellular localization of LOX proteins and LOX activity was examined in olive cotyledons by using fluorescence and light microscopy, respectively. In mature seeds, the fluorescent labelling was detected in the area of PBs (Fig. 4A). Purple colour, indicating LOX activity, was visible mainly in PBs (Fig. 4A, inset). After seed imbibition, the intensity of the fluorescence and of LOX activity staining increased in PBs as well as in the cytoplasm surrounding OBs (Fig. 4B and inset). After 6h of *in vitro* germination, high fluorescent labelling was observed in the area of PBs and cytoplasm (Fig. 4C). At this stage, significant LOX activity was visible on the boundary of OBs (Fig. 4C, inset). After 3 d of *in vitro* germination, both LOX proteins and activity were found in numerous, rounded areas located in the matrix of the PBs (Fig. 4D, circles). Moreover, intense LOX activity was attached to the boundary of OBs (Fig. 4D, inset). After 4 d of seedling growth, LOX antigen was located inside large PBs and in the surrounding cytoplasm (Fig. 4E). Weak LOX activity was visible in the area of PBs and cytoplasm (Fig. 4E, inset). After 15 d, when a large central vacuole was formed, the fluorescent labelling and LOX activity were detected mainly in the cytoplasm surrounding the central vacuole as well as in the chloroplasts (Fig. 4F and inset). Control reactions did not show any labelling (Supplementary Fig. S1B–D at *JXB* online).

**Fig. 5.**
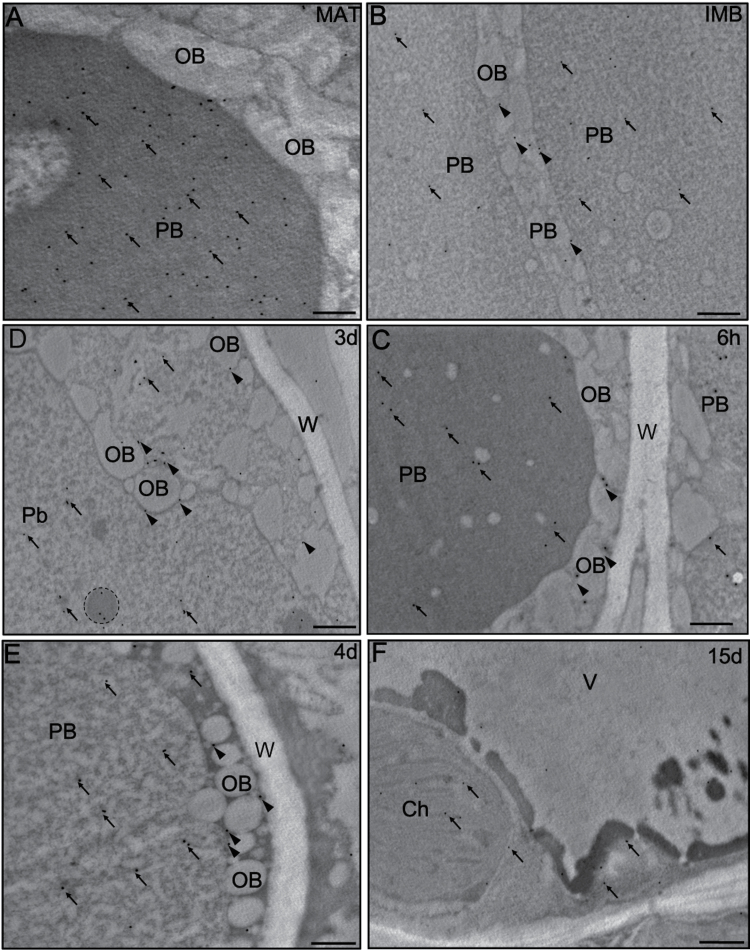
(A–F) Ultrastructural localization of LOX in olive cotyledons. Gold particles are located on the OB boundaries (arrowheads) and in the PBs (arrows). Some labelling is also present in the surrounding cytoplasm and chloroplasts (arrows). Ch, chloroplast; OB, oil body; PB, protein body; W, cell wall. Bars=1 μm.

Gold immunolabelling of LOX proteins provided additional details about its subcellular localization, and confirmed the tissue distribution pattern observed by fluorescence microscopy. In the mature seed, the gold particles were located in the area of PBs (Fig. 5A). After seed imbibition, the labelling was found in the area of PBs (Fig. 5B, arrows) as well as on the boundaries of OBs (Fig. 5B, arrowheads). A similar pattern of labelling was observed 6h after imbibition (Fig. 5C). After 3 d of *in vitro* germination, the gold particles were detected in the electron-dense areas located in the PB matrix (Fig. 5D, circle). In the large PBs formed after 4 d of seedling growth, gold particles were found in the area of the PBs (Fig. 5E, arrows) and OBs (Fig. 5E, arrowhead). As cotyledon development progressed (15th day), the signal was localized in the chloroplasts as well as in the surrounding cytoplasm (Fig. 5F, arrows). Control reactions did not show any gold labelling (Supplementary Fig. S1E, F at *JXB* online).

### Cellular localization of lipase and PLA activities in living olive cotyledons

**Fig. 6.**
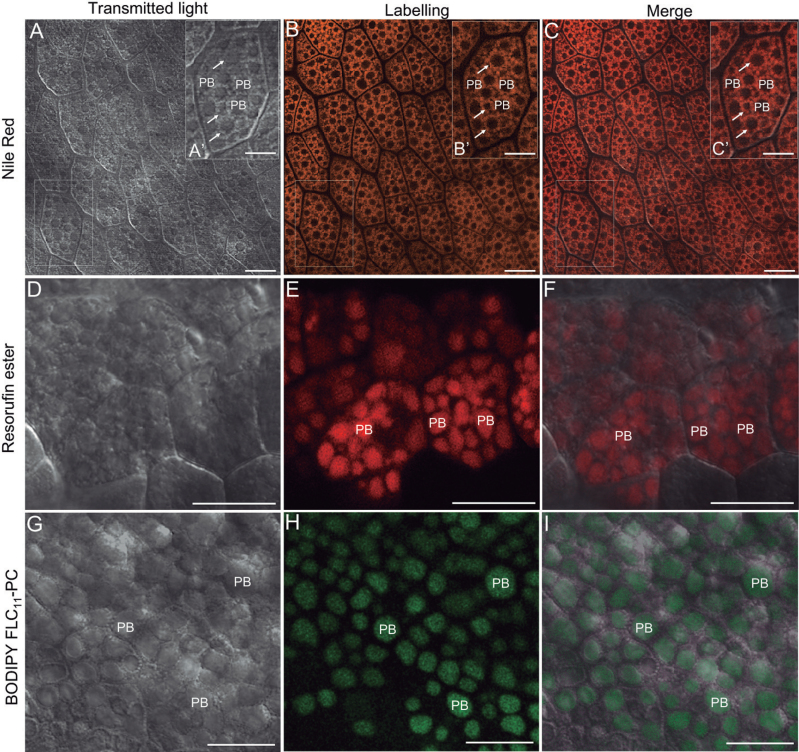
Localization of OB lipase (resorufin ester) and phospholipase A (BODIPY^®^ FL C_11_-PC) activities in living olive cotyledons dissected out from mature seed by confocal laser scanning microscopy. (A–C) Olive cotyledons stained with Nile Red. Numerous OBs are indicated with arrows. (D–F) Olive cotyledons incubated with resorufin ester. Lipase activity is located mainly in the area of PBs. (G–I) Olive cotyledons incubated with BODIPY^®^ FL C_11_-PC. Green fluorescent labelling is detected exclusively in the PB area. PB, protein body. Bars=25 μm, inset bars=10 μm.

**Fig. 7.**
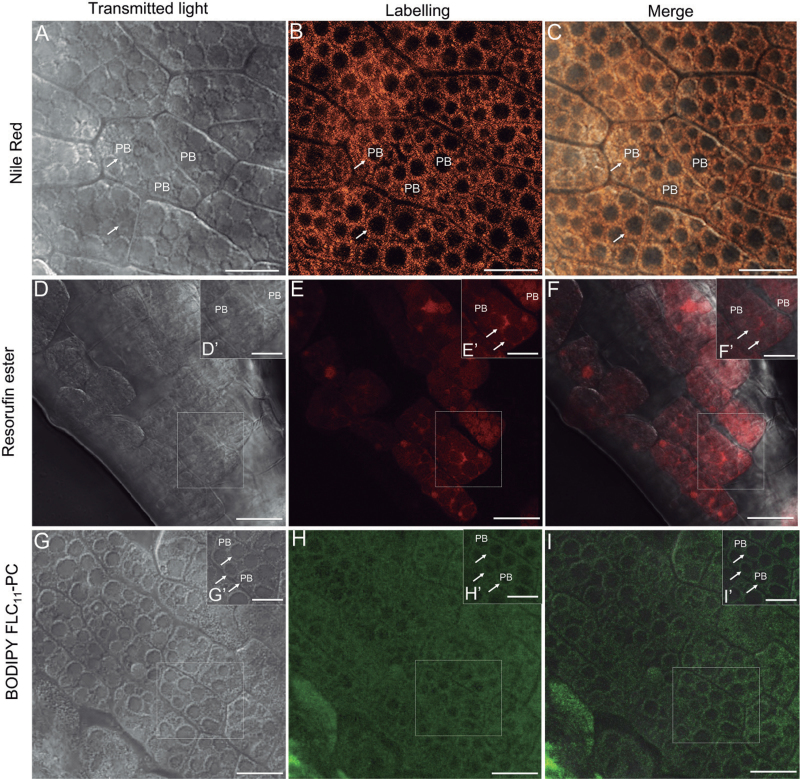
Localization of OB lipase (resorufin ester) and phospholipase A (BODIPY^®^ FL C_11_-PC) activities in living olive cotyledons dissected out from imbibed seed by confocal laser scanning microscopy. (A–C) Optical section of olive cotyledons stained with Nile Red. Numerous OBs are indicated with arrows. (D–F) Olive cotyledons incubated with resorufin ester. Lipase activity is detected in the area of PBs as well as on the OB surface (arrows). (G–I) Olive cotyledons incubated with BODIPY^®^ FL C_11_-PC. Green fluorescent labelling was detected only on the OB surface (arrows). PB, protein body. Bars=25 μm, inset bars=10 μm.

The viability of the cotyledon cells, their intact structure, and the integrity of their membranes were confirmed by using FM4-64 dye and SYTOX green (Supplementary Figs S2, S3 at *JXB* online). After Nile Red staining, the OBs in olive cotyledon cells were visible as numerous red-orange fluorescence spots surrounding non-labelled PBs (Figs 6A–C, 7A–C). *In situ* lipase activity was detected by using a lipase substrate (resorufin ester). The product of its hydrolysis emitted red fluorescence after excitation with light of 544nm (Figs 6D–F, 7D–F). BODIPY^®^ FL C_11_-PC was used for detection of PLA activities. The cleavage product generated from this substrate by PLAs can be detected as green fluorescence under the wavelength of 488nm (Figs 6G–I, 7G–I). In the cotyledons from mature, dry seed, the lipase activity was observed exclusively in the area of PBs (Fig. 6D–F). A similar localization pattern was found for products of PLA activity (Fig. 6G–I). After seed imbibition, the lipase activity was located inside PBs as well as in the area occupied by OBs (Fig. 7D–F). The green fluorescence, indicating PLA activity, was located exclusively in the area occupied by OBs (Fig. 7G–I, arrows). No fluorescent labelling was found in PBs.

### Detection of oil body-associated enzymes in olive cotyledons

**Fig. 8.**
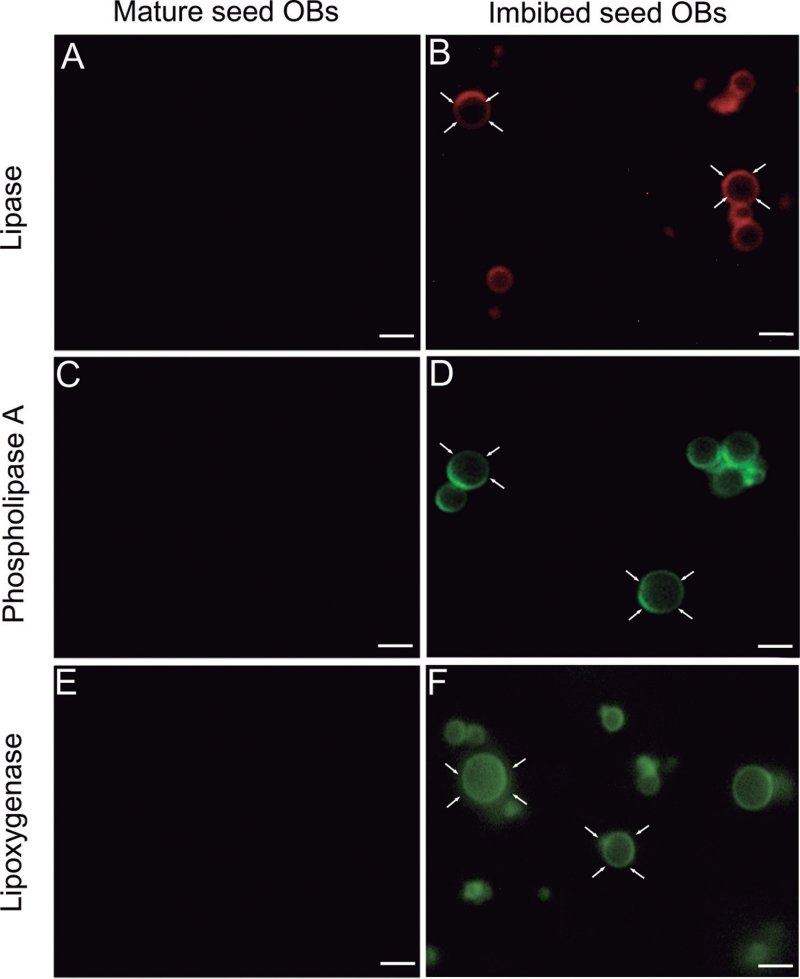
Detection of lipase (resorufin ester) and phospholipase A (BODIPY^®^ FL C_11_-PC) activity and lipoxygenase (anti-LOX Ab) on OBs isolated from cotyledons dissected out from mature and imbibed seed. (A and B) Lipase activity visible as red fluorescence is located on the OB surface in imbibed seed (arrows). OBs isolated from mature seed do not show any labelling. (C and D) Green fluorescence is detected only on the surface of OBs (arrows) isolated from cotyledons after seed imbibition. (E and F) LOX is detected on the surface of OBs (arrows) in imbibed seed but it is absent in OBs isolated from cotyledons of mature seed. Bars=10 μm.

Purified OBs were visible as spherical structures (0.2–2 μm) that stained positively with Nile Red (Supplementary Fig. S4 at *JXB* online). The purity of isolated OBs was confirmed by the absence of ubiquitin (cytoplasmic marker) on their surface (Supplementary Fig. S5A, B). Additionally, the identity of isolated OBs and the integrity of their membrane were verified by co-localization of LOX and caleosin (Supplementary Fig. S5C–E). OBs isolated from cotyledons at seed maturity did not show the presence of lipase activity (Fig. 8A). After imbibition, a strong lipase activity appeared on the surface of the OBs as ring-shaped red fluorescence (Fig. 8B). The activity of PLAs was detected only on the surface of OBs isolated from imbibed seeds (Fig. 8C, D). The presence of LOX protein on the surface of OBs was also tested. The LOX was detected only on the surface of OBs isolated from cotyledons of imbibed seeds, but it was absent in OBs isolated from mature, dry seed (Fig. 8E, F).

## Discussion

In germinating oilseeds, such as *Arabidopsis* or cucumber, neutral lipids stored in OBs are mobilized to provide the carbon skeletons and energy necessary for their post-germinative growth (Graham, 2008). This process occurs by the action of hydrolytic enzymes such as phospholipase, LOX, and lipase at different stages of seed germination (Rudolph *et al.*, 2011). Here, the changes in the *in vivo* localization of PLA activity in cotyledons during seed imbibition are reported for the first time. Interestingly, the PLA activity in the cotyledons from mature olive seed was associated with PBs, while after seed imbibition PLA activity was evident in the cytoplasm surrounding the OBs and on their surface. To date PLA activity had been detected only on the OB surface and in the cytoplasm of sunflower cotyledon protoplasts. PLA was expressed or activated on the surface of OBs during the early phase of seedling development, prior to the action of lipase (Bhatla *et al.*, 2009). In the light of the microscopy findings reported here, PLA activity in the olive cotyledons could be shifted from PBs to OBs concomitantly with the progress of seed germination. Moreover, a close temporal correlation was found between PLA and lipase activities during olive seed germination. These data suggest that PLA activity associated with the surface of OBs is responsible for the phospholipid breakdown leading to TAG mobilization by lipase.

Lipase is major enzyme associated with the seed (Eastmond, 2006; Kelly *et al.*, 2011). Lipase is known to be associated with the membranes of OBs, glyoxysomes, and with PBs (Fernandez and Staehelin, 1987, Beisson *et al.*, 2001; Hayashi *et al.*, 2001). Using α-naphthyl palmitate as an enzyme substrate, a group ofseven putative lipases were identified in olive cotyledons. Interestingly, only 53kDa lipase activity was present in PBs during the early steps of seed germination. The lipase activity was detectable in PBs mainly in the areas where lipids were present. Indeed, some studies suggested that lipase could be initially localized at its storage sites in the PBs or PSVs (Fernandez and Staehelin, 1987; Bhatla *et al.*, 2009). Moreover, it was demonstrated that OBs interact with the PSV tonoplast in the germinating *Arabidopsis* seed, and this interaction might be promoted by caleosin of the OBs (Poxleitner *et al.*, 2006). These data suggest that the interaction of OBs with the membrane of another organelle could help breach the phospholipid monolayer of the OB, providing access to lipase. The present results indicate that lipase present in PBs could act by facilitating the metabolism of storage lipids in PBs during seed germination. After using α-naphthyl palmitate as the lipase substrate, lipase activity was not shown on the surface of the OBs. However, the presence and activity of an alternative lipase responsible for OB hydrolysis cannot be ruled out. A previous study described evidence of a 95kDa patatin domain TAG lipase (SDP1) associated with purified OBs and involved in their mobilization in germinating *Arabidopsis* seeds (Eastmond, 2006). In order to detect TAG lipase activity in olive OBs, resorufin ester was used as a marker for lipase intracellular localization. Lipase activity was initially localized in PBs prior to seed germination, this activity becoming progressively transferred to the OB surface during early steps of olive seed germination. This pattern was also observed in sunflower cotyledon protoplasts, where lipase activity shifted from the PSVs to OBs during seed germination (Bhatla *et al.*, 2009). The different substrate-dependent localization patterns of lipase activity in olive cotyledons suggest that some lipases may act only in PBs, whereas others may hydrolyse TAGs in both PBs and OBs.

Fatty acids released from TAGs may be further degraded either via the β-oxidation in glyoxysomes or by an alternative pathway, which is dependent on a LOX enzyme (Feussner *et al.*, 2001; Graham, 2008). A recent study found evidence that the lipid breakdown by LOX in cucumber cotyledons is promoted by a patatin-like phospholipase (Rudolph *et al.*, 2011). The anti-LOX Ab used in this work recognized two protein bands, of 98kDa and 100kDa, on western blots. However, the LOX activity profile revealed the existence of a third LOX isoenzyme with an estimated mol. wt of 96kDa. The sizes of olive cotyledon LOXs are well within the molecular weight range (95–100kDa) reported for other plant LOXs (Andreou and Feussner, 2009). The present study also detected a smaller protein band of 63kDa, which showed enhanced LOX activity. In a previous study (Zienkiewicz *et al.*, 2013) a 63kDa protein band with LOX activity was also detected in olive pollen. Interestingly, limited proteolysis of soybean LOX1 generated a 60kDa fragment with enhanced activity and membrane binding ability (Maccarrone *et al.*, 2001). Although LOX expression and activity subsequently increased during the initial steps of *in vitro* germination, a further decrease occurred coincidentally with the reduction in the number of OBs (Zienkiewicz *et al.*, 2011) in the cytoplasm of olive cotyledons. A similar pattern was also observed in barley embryos, in which levels of LOX expression and activity declined during the last phase of germination (Holtman *et al.*, 1996). Olive LOXs were mainly localized in PBs as well as on the surface of OBs. Here, the presence of LOXs in the PBs during *in vitro* germination was detected for the first time. The localization of LOXs in both the cytoplasm and PBs suggests that this enzyme may be shifted from PBs to the cytoplasm and the OB surface. Interestingly, LOXs were specifically localized in rounded intra-PB areas. A previous study reported that these areas do not contain SSPs but do contain peroxidase (POX) (Zienkiewicz *et al.*, 2011). It has been showed that fatty acid peroxides, which are products of LOX activity, subsequently serve as substrates for POX (Koljak *et al*., 1997; Brash, 1999). A functional relationship between LOX and POX has been previously studied in developing oat plants at four stages: germination, growth, senescence, and dark-incubated senescence (Yi *et al.*, 2005). LOX and POX were found to exhibit similar activity patterns in all examined stages (Yi *et al.*, 2005). Specific co-localization of LOXs and POX in PBs of olive cotyledon seems to confirm this functional relationship.

## Conclusions

PBs from olive cotyledons store PLA and lipase at seed maturity, which are transferred to the cytoplasm and the surface of OBs during early steps of germination. Meanwhile, the activity of neutral lipases is limited mainly to PBs and their distribution is closely correlated with the appearance of neutral lipids in the PB area as germination progresses. LOX enzymes act both in PBs and on the surface of OBs during the initial phase of seed germination. At this time, OB-associated LOX activity is temporally correlated with the appearance of PLA and lipase activities on the OB surface. To the authors’ knowledge, this is the first report indicating the presence of diverse lipolytic enzymes inside the PBs implicated in the mobilization of storage lipids during seed germination. It is proposed that PBs should be considered not only as simple storage structures but also as dynamic and multifunctional organelles.

## Supplementary data

Supplementary data are available at *JXB* online.

Figure S1. Negative controls.

Figure S2. Fluorescent labelling of cellular membranes with FM4-64 dye in the olive cotyledon cells.

Figure S3. Analysis of cell viability in fresh cotyledon tissue by using SYTOX green.

Figure S4. Purified oil bodies (arrows) stained with Nile Red and observed under transmitted light (A) and under fluorescence (B).

Figure S5. Localization of ubiquitin on isolated OBs (A) using the anti-UBQ Ab, showing the absence of ubiquitin (cytoplasmic marker) on their surface (B). Co-localization of caleosin (C) and LOX (D) on isolated OBs using the anti-Clo3 and anti-LOX Abs (E).

Table S1. Gene accession numbers and sequences of the primers used in this study.

Supplementary Data
