# Supplementary material for: Olive seed protein bodies store degrading enzymes involved in mobilization of oil bodies
Source: J Exp Bot. 2013 Oct 29;65(1):103–15. doi: 10.1093/jxb/ert355 (PMC3883284; doi:10.1093/jxb/ert355)
Supplement: Supplementary Data [file supp_65_1_103__index.html]

Olive seed protein bodies store degrading enzymes involved in mobilization of oil bodies — Olive seed protein bodies store degrading enzymes involved in mobilization of oil bodies — Supplementary Data 

# Olive seed protein bodies store degrading enzymes involved in mobilization of oil bodies

## Supplementary Data

Data files

**Files in this Data Supplement:**

- Supplementary Data - Supplementary Data
