# Supplementary material for: Olive seed protein bodies store degrading enzymes involved in mobilization of oil bodies
Source: J Exp Bot. 2013 Oct 29;65(1):103–15. doi: 10.1093/jxb/ert355 (PMC3883284; doi:10.1093/jxb/ert355)
Supplement: Supplementary Data [file supp_ert355_jexbot101717_file001.pdf]

Olive seed protein bodies store degrading enzymes involved in oil bodies mobilization

Agnieszka Zienkiewicz, Krzysztof Zienkiewicz, Juan David Rejón, Juan de Dios Alché, Antonio Jesús Castro, María Isabel Rodríguez-García

Table S1. Gene accession numbers and sequences of the primers used in this study.

Gene	Accession number	Sequence
OeLOX1	EU513351	Forward: 5’-GAAAACAATTACCGCCCAGTTCCA-3’<br>Reverse: 5’-TTTATCCGAAGGCCACTCAC-3’
OeLOX2	EU513353	Forward: 5’-GTTCGCTGGGAAAGTGAAAGAG-3’<br>Reverse: 5’-TCAAATGGAAACGCTGTTAGG-3’
OeUBQ2	AF429430	Forward: 5’-AATGAAGTCTGTCTCTCCTTTGG-3’<br>Reverse: 5’-AAGGGAAATCCCATCAACG-3’

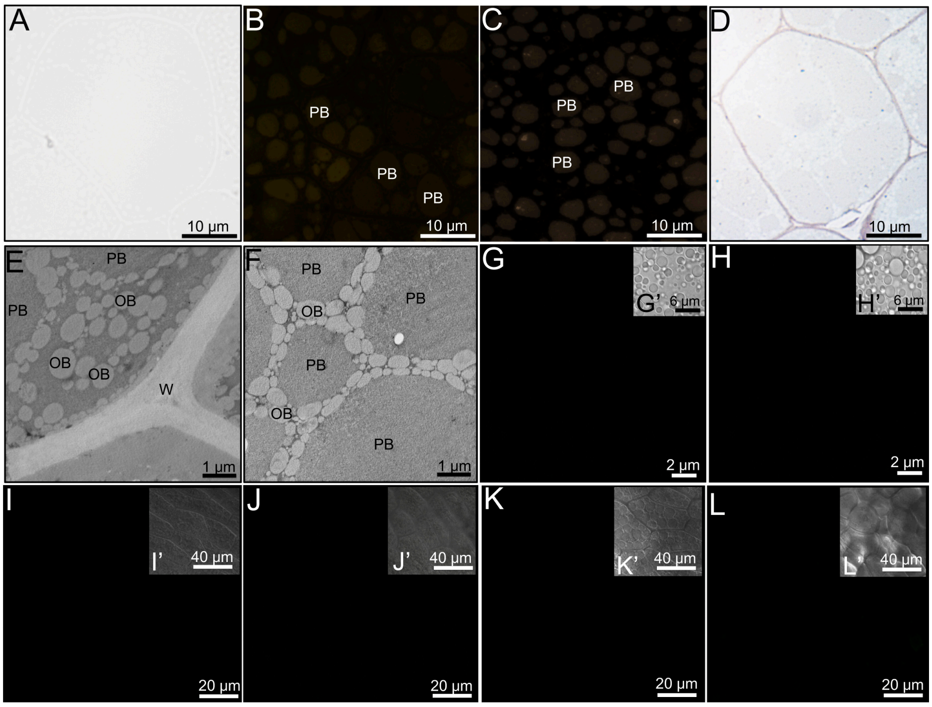

Fig. S1. (A) Negative control for the detection of lipase activity generated by omitting the α-naphthyl palmitate in the reaction mixture. (B) Negative control for LOX immunofluorescence localization prepared by omitting the primary Ab. (C) Negative control for LOX immunofluorescence localization performed with pre-immune rabbit serum. (D) Negative control for the detection of lipoxygenase activity prepared by omitting α-linolenic acid in the reaction mixture. (E) Negative control for LOX immunogold localization carried out by omitting the primary Ab. (F) Negative control for LOX immunogold localization prepared with pre-immune rabbit serum. (G) Negative control performed by omitting the incubation of isolated OBs with primary Abs. (H) Negative control prepared by incubation of OBs with the pre-immune rabbit serum. (I) Negative control for the cell analysis of lipase activity prepared by omitting the resorufin ester substrate in the reaction mixture. (J) Negative control for the cellular localization of phospholipase A activity generated by omitting the BODIPY® FL C_{11} -PC substrate in the reaction mixture. (K) Negative control for the analysis of cellular membrane localization carried out by omitting the FM4-64 dye in the reaction mixture. (L) Negative control for the analysis of viability of the cotyledon cells carried out by omitting the SYTOX green in the reaction mixture. PB, protein body; OB, oil body; W, cell wall.

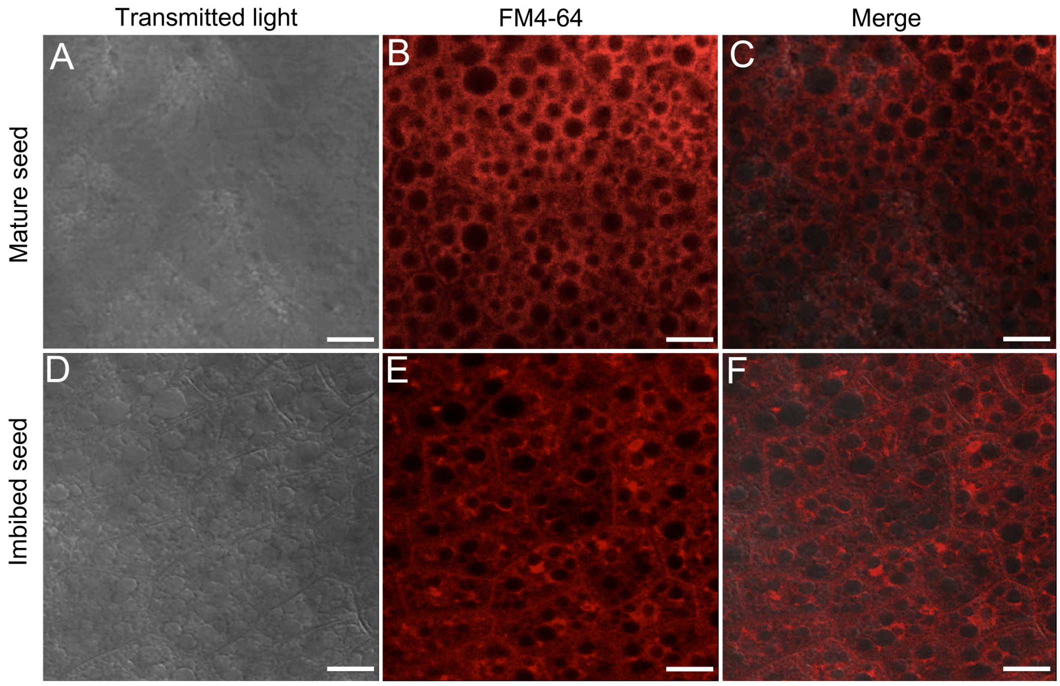

Fig. S2. Fluorescent labeling of cellular membranes with FM4-64 dye in the olive cotyledons cells. (A-C) Mature seed. (D-F) Imbibed seed. Bars=25 µm.

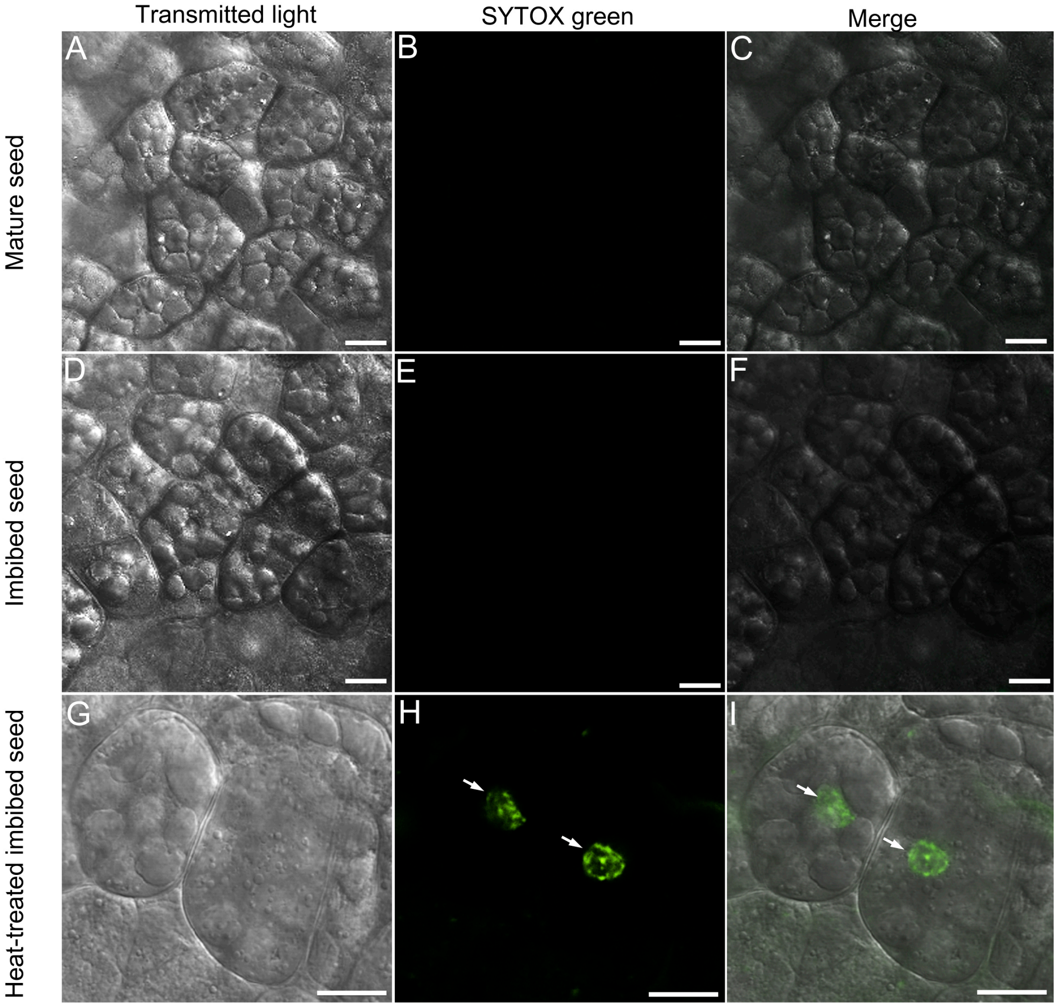

Fig. S3. Analysis of cells viability in fresh cotyledon tissue by using SYTOX green. (A-C) Mature seed. (D-F) Imbibed seed. (G-I) Imbibed seed treated with heat. Stained nucleic acids are marked by arrows. Bars=10 μm.

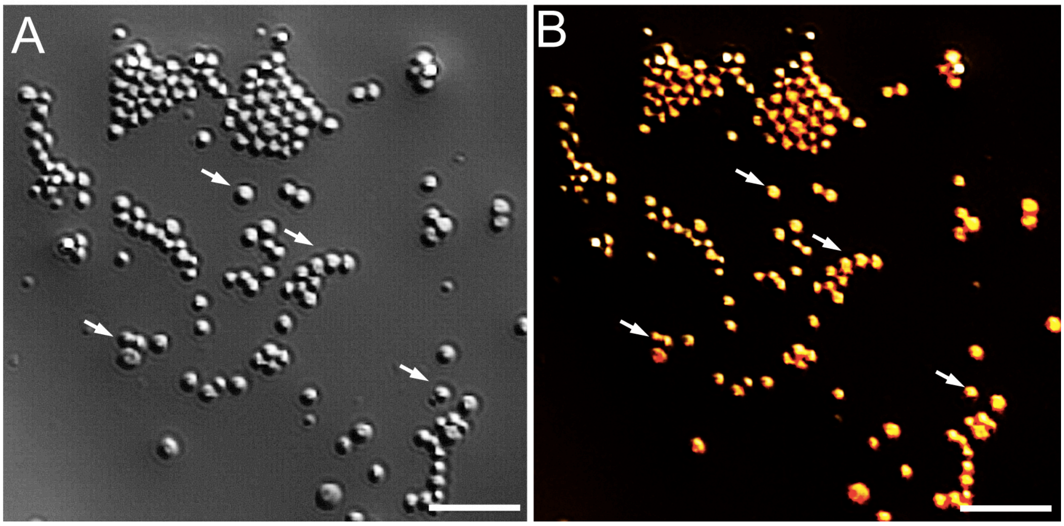

Fig. S4. Purified oil bodies (arrows) stained with Nile Red and observed under transmitting light (A) and under fluorescence (B). Bars=10 μm.

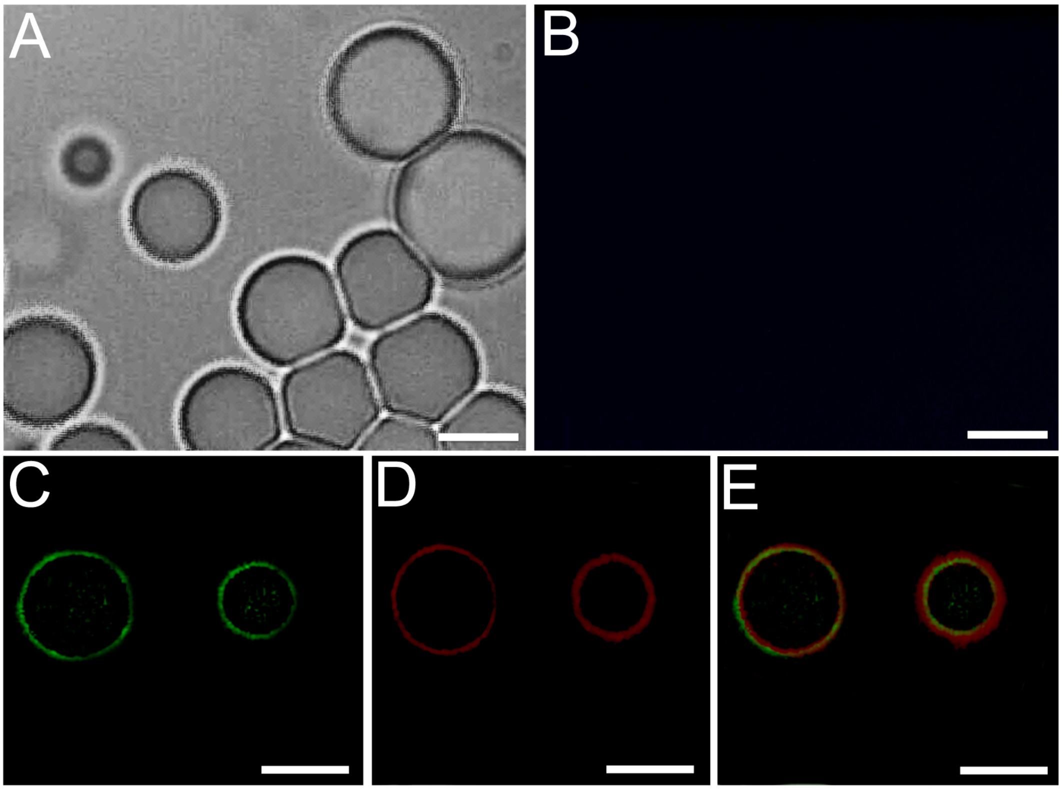

Fig. S5. Localization of ubiquitin on isolated OBs (A) using the anti-UBQ Ab, showing the absence of ubiquitin (cytoplasmic marker) on their surface (B). Co-localization of caleosin (C) and LOX (D) on isolated OBs using the anti-Clo3 and anti-LOX Abs (E). Bars=2 μm.
